# Urothelial Dysfunction and Increased Suburothelial Inflammation of Urinary Bladder Are Involved in Patients with Upper Urinary Tract Urolithiasis – Clinical and Immunohistochemistry Study

**DOI:** 10.1371/journal.pone.0110754

**Published:** 2014-10-20

**Authors:** Yuan-Hong Jiang, Hann-Chorng Kuo

**Affiliations:** Department of Urology, Buddhist Tzu Chi General Hospital and Tzu Chi University, Hualien, Taiwan; Universidade de Sao Paulo, Brazil

## Abstract

**Objectives:**

To investigate the urothelial dysfunction and inflammation of urinary bladder in patients with upper urinary tract (UUT) urolithiasis through the results of cystoscopic hydrodistension and immunohistochemistry study.

**Methods:**

Ninety-one patients with UUT urolithiasis underwent cystoscopic hydrodistension before the stone surgery. Immunofluorescence staining of E-cadherin, zonula occludens-1 (ZO-1), tryptase (mast cell activation), and TUNEL (urothelial apoptosis) were performed in 42 patients with glomerulations after hydrodistension, 10 without glomerulations, and 10 controls.

**Results:**

Of the 91 patients, 62 (68.2%) developed glomerulations after hydrodistension. Lower urinary tract symptoms (LUTS) were present in 53.8% patients, in whom significantly smaller maximal anesthetic bladder capacity (MBC) was noted. Patients with middle or lower 1/3 ureteral stones had a significantly higher glomerulation rate (88.6% vs. 55.4%, p<0.01) and lower MBC (618.4±167.6 vs. 701.2±158.4 ml, p = 0.027) than those with upper 1/3 ureteral or renal stones. Patients with UUT urolithiasis had significantly lower expression of E-cadherin (26.2±14.8 vs. 42.4±16.7) and ZO-1 (5.16±4.02 vs. 11.02±5.66); and higher suburothelial mast cell (13.3±6.8 vs. 1.3±1.2) and apoptotic cell (2.6±2.5 vs. 0.1±0.3) numbers than in controls (all p<0.01).

**Conclusions:**

Urothelial dysfunction and increased suburothelial inflammation and apoptosis are highly prevalent in the bladders of UUT urolithiasis patients, indicating inflammation cross-talk between UUT and urinary bladder. Patients with UUT urolithiaisis concomitant with LUTS had a smaller MBC, which may explain the presence of irritative bladder symptoms.

## Introduction

Patients with upper urinary tract (UUT) urolithiasis, including renal and ureteral stones, often have lower urinary tract symptoms (LUTS), including urinary frequency, urgency, and dysuria mimicking cystitis, especially in patients with stones in the lower 1/3 ureter. Similar innervation of ureter and bladder is a probable explanation, but the communication between UUT and bladder is not fully understood in human subjects. The pathomechanisms of interstitial cystitis/bladder pain syndrome (IC/BPS) include neurogenic inflammation, urothelial dysfunction, activation of mast cells, autoimmunity, and occult infection. [1]–[3] It is unclear whether neural mechanisms and inflammation are the causes or results of the initiating events in IC/BPS, but neural up-regulation is known to play a role in the chronicity of pain, urgency, and frequency. [4] In addition, impaired urothelial homeostasis with increased suburothelial inflammation and apoptosis were demonstrated in the bladders of patients with IC/BPS. [3].

In our unpublished comparative study of bladder glomerulations in patients with IC/BPS and the other lower urinary tract diseases including stress urinary incontinence, benign prostate obstruction, overactive bladder, and UUT urolithiasis, a significantly higher incidence of glomerulations in UUT urolithiasis patients was observed after cystoscopic hydrodistension. Therefore, we speculated that inflammation in the bladder wall with impaired urothelial homeostasis might involve not only the pathogenesis in IC/BPS but also UUT urolithiasis. The purposes of this study were to investigate the urothelial dysfunction and bladder inflammation in patients with UUT urolithiasis, and to prove the essentiality of exclusion of UUT urolithiasis in the diagnosis of IC/BPS through the results of cystoscopic hydrodistension and immunohistochemistry study.

## Materials and Methods

### Patients and Procedures

From May 2012 to April 2013, patients who had UUT urolithiasis that required surgical intervention in a tertiary teaching hospital were prospectively and consecutively enrolled in the study. Exclusion criteria included the patients with the history of spinal cord injury with neurogenic voiding dysfunction, IC/BPS, overactive bladder, urinary tract malignancy, proven bladder outlet obstruction, or recurrent urinary tract infection. Eligible patients were grouped into patients with stone positions in the middle or distal ureter and those with stone positions in the upper ureter or kidney.

All patients were admitted for the surgical procedure including uretero-renoscopic stone manipulation or percutaneous nephrolithotripsy. Patients with symptomatic urinary tract infection, or pyuria as well as bacteriuria in urinalysis and/or a positive urine culture on admission were excluded from this study. Pyuria was defined as more than 10 white blood cells per high power field in urinalysis. Cystoscopic hydrodistension was performed under general anesthesia before the stone surgery. The maximal anesthetic bladder capacity (MBC) was recorded during cystoscopic hydrodistension at the intravesical pressure of 80 cmH_2_O, and glomerulation hemorrhage after hydrodistension was recorded as grade 0 to 4 representing none, mild, moderate, and severe. [9] Of the 91 eligible patients, 52 agreed to receive bladder wall biopsies after hydrodistension. Cold cup bladder biopsies were obtained from the posterior wall about 2cm above the ureteral orifice, and only the bladder mucosa and submucosa were taken to prevent bladder perforation. One bladder specimen was sent to the pathology department for hematoxylin and eosin staining to exclude the possibility of carcinoma in situ or malignancy. The other specimens were embedded in OCT (optimum cutting temperature) medium and stored frozen at −80C for later immunofluorescence staining. For comparison, bladder biopsies from 10 patients with stress urinary incontinence but without LUTS (serving as controls) were taken after anti-incontinence procedures and cystoscopic hydrodistension.

This study was approved by the Institutional Review Board and Ethics Committee of Buddhist Tzu Chi General Hospital. Each patient was informed about the study rationale and procedures, and written informed consent was obtained before the bladder procedures.

### Immunofluorescence Staining and Quantification of Protein Expression

Bladder tissues from urolithiasis patients and controls were investigated for urothelial adhesive function by E-cadherin expression, urothelial integrity by zonula occludens-1 (ZO-1, a junction protein) expression, mast cell activation by tryptase level, and suburothelial and urothelial apoptosis by terminal deoxynucleotidyl transferase dUTP nick end labeling (TUNEL) assay. Laboratory procedures were in the similar manner as our previous studies. [5], [6].

The urinary bladder specimens were immersed and fixed for 1h in an ice-cold solution of 4% formaldehyde in phosphate buffered saline (PBS, pH 7.4). They were then rinsed with ice-cold PBS containing 15% sucrose for 12h. Biopsy specimens were embedded in OCT medium and stored at −80C. Four sections per specimen were cut using a cryostat at a thickness of 8 µm and collected on new silane III-coated slides (Muto Pure Chemicals Co. Ltd, Tokyo, Japan). Sections were post-fixed in acetone at −20C and blocked with rabbit serum. The sections were incubated overnight at 4C with primary antibodies to anti-human E-cadherin (BD Biosciences, Franklin, NJ, USA), anti-human ZO-1 (Invitrogen, Burlington, ON, Canada) or anti-human mast cell tryptase (Chemicon, Temecula, CA, USA). After rinsing the sections with 0.1% Tween-20 in PBS, rabbit anti-mouse conjugated fluorescein isothiocyanate secondary antibodies (DakoCytomation, Denmark A/S) were applied to the sections and incubated for 1h. Finally, the sections were counterstained with diamidino-2-phenylindole (DAPI) (Sigma Chemical Co., St. Louis, MO, USA). Negative controls included the isotype of the primary antibody.

The sections were incubated with 100 µL 20 µg/mL proteinase K (Calbiochem, Darmstadt, Germany) at room temperature for 20min and washed with PBS. The sections were covered with 100 µL of terminal deoxynucleotidyl transferase (TdT) equilibration buffer (Calbiochem) and incubated at room temperature for 30min. After carefully blotting the 1X TdT equilibration buffer from the specimens, we applied TdT labelling reaction mixture (Calbiochem) onto the specimens and incubated them for 90min at 37C. The positive control, apoptotic HL-60 cells (Calbiochem), was treated with 1 µg/µL DNase I in Tris buffered saline/1 mM MgSO4 (Promega Corp., Madison, WI, USA) at room temperature for 20min. The negative control was generated by substituting dH2O for the TdT enzyme in the reaction mixture. After washing with PBS, the cells were mounted using Fluorescein-FragELTM mounting medium (Calbiochem). The total cell population was visualized using a 330 to 380-nm filter for DAPI while the analysis labelled nuclei were visualized using a standard fluorescein filter (465–495 nm).

The results of immunofluorescence (tryptase, and TUNEL) were quantified by counting the average positive cells/total cells per unit area (4 µm^2^) of 3 high-power fields in the area with the greatest infiltrate density, and were shown as the percentage of positive cells per 100 total cells. The intensities of E-cadherin and ZO-1 were quantified using Image J processing. [7] All procedures were performed by technicians who were blind to the patient allocation.

### Statistical Analysis

Continuous variables were presented as means ± standard deviations, and categorical data were presented as numbers and percentages. Eligible patients were grouped according to the stone position; the data for MBC and glomerulation hemorrhage during hydrodistension were compared. Differences in expression of E-cadherin and ZO-1, mast cell activation, and suburothelial and urothelial apoptosis between urolithiasis and the control bladder tissues were analyzed using the Kruskal-Wallis test. All calculations were performed using SPSS for Windows, version 16.0 (SPSS, Chicago, IL). P value of less than 0.05 was considered significant.

## Results

The study included 56 men and 35 women with UUT urolithiasis. Their mean age was 55.4±13.4 years. After cystoscopic hydrodistension, the mean MBC was 668.9±166.1 ml, and 62 patients (68.1%) developed glomerulation hemorrhage. Among these patients, most glomerulations were grade I (47 of 62 patients, 75.8%). Although diffused glomerulations were found in some patients, no bladder ulcer was found (Table 1). Male patients had a smaller MBC than female patients (639.8±151.2 vs 718.5±173.4 ml, p = 0.032), but their glomerulation rates were similar (64.3 vs 74.5%, p = 0.437). LUTS were present in 53.8% of patients, including frequency, urgency, nocturia, dysuria, and residual urine sensation. Patients with LUTS had a significantly smaller MBC than those without LUTS (610.7±145.7 vs 718.8±76.1 ml, p = 0.012).

**Table 1:** Demographic and cystoscopic hydrodistention data from patients with upper urinary tract urolithiasis.

	Middle or lower 1/3 ureteral stones	Upper 1/3 ureteral or renal stones	Total	P value
N	35	56	91	
Gender	25 M, 10 F	31 M, 25 F	56 M, 35 F	0.094
Age (year)	53.3±14.8	56.8±12.3	55.4±13.4	0.232
MBC (ml)	618±168	701±158	669±166	0.027
Glomerulation*	88.6% (31)	55.4% (31)	68.1% (62)	0.001
Grade 1	77.4% (24)	74.2% (23)	75.8% (47)	0.520
Grade 2	19.4% (6)	25.8% (8)	22.6% (14)	
Grade 3	3.2% (1)	0	1.6% (1)	

*Data given as % (n) unless otherwise indicated.

UUT: upper urinary tract; MBC: maximal anesthetic bladder capacity.

Of the 91 patients, 35 patients had stones in the middle or lower 1/3 ureter, and 56 had stones in upper 1/3 ureter or kidney. In patients with middle or lower 1/3 ureteral stone, the glomerulation rate was significantly higher than in those with upper 1/3 ureteral or renal stones (88.6% vs 55.4%, p = 0.001), but the severity of glomerulations was similar between two groups (p = 0.520). Patients with middle or lower 1/3 ureteral stone had a significantly smaller MBC (618.4±167.6 vs 701.2±158.4 ml, p = 0.027) than those with upper 1/3 ureteral or renal stones. Twenty-three patients with renal stones had a mean MBC of 678.6±187.5 ml and 56.5% glomerulation rate. Within each group, there was no significant difference in MBC or glomerulation rate between sex.

**Figure 1:**
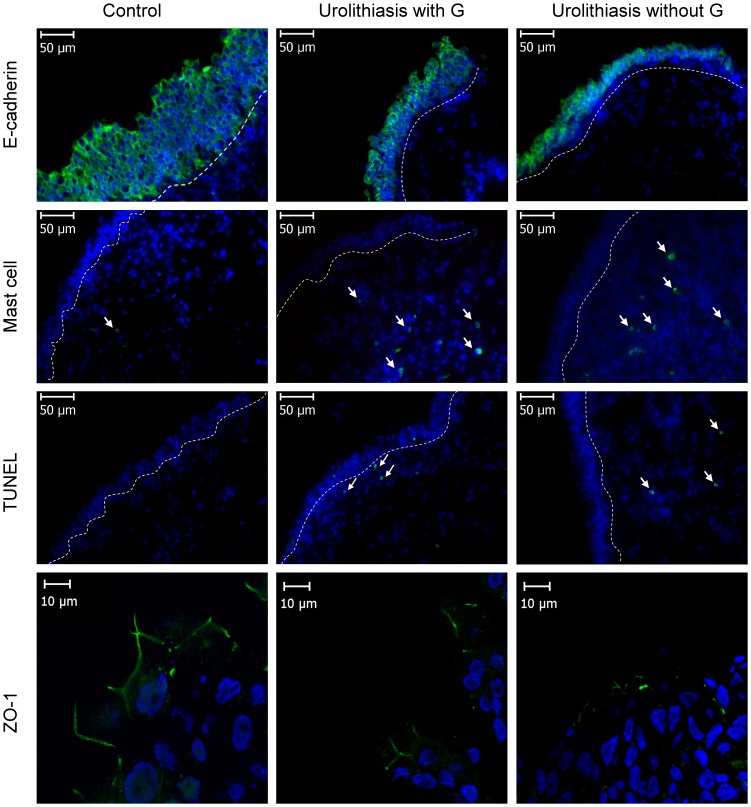
Immunofluorescence staining of adhesive protein E-cadherin expression, junction protein ZO-1 expression, mast cell counts and apoptotic cell counts in the bladder tissues of upper urinary tract urolithiasis patients with or without glomerulation (G) and controls. The target cells are labeled green, and the white dotted line indicates the boundary between the urothelium and suburothelium.

Of the 91 eligible patients, 52 agreed to undergo bladder wall biopsies after cystoscopic hydrodistension. In immunofluorescence staining, the expression of E-cadherin and ZO-1 in the bladder urothelium of patients with UUT urolithiasis was significantly lower than that of controls (p<0.001 and p = 0.003, respectively) (Figure 1, table 2). More activated mast cells in the suburothelium were observed in the UUT urolithiasis group than in the control group (13.3±6.8 vs 1.3±1.2, p<0.001). In TUNEL staining, the apoptotic cell counts were significantly higher in the UUT urolithiasis group than in the control group (2.6±2.5 vs 0.1±0.3, p = 0.002). Despite the presence of glomerulations, LUTS, or different stone positions, we had found no significant difference in their immunohistochemistry findings (all p>0.05) (Table 2). Within these patients, there was no significant difference in immunofluorescence analysis between sex except activated mast cell numbers (male 14.8±6.7 VS female 10.7±5.9, p = 0.029).

**Table 2:** Immunofluorescence analysis of bladder tissues from patients with upper urinary tract urolithiasis and controls.

	UUT Urolithiasis					Control	P value
	G (+)	G (−)	Lower UUT	Upper UUT	Total		
N	42	10	20	32	52	10	
Age (year)	53.4±13.1	60.6±11.5	53.1±15.3	55.7±11.4	54.9±13.0	50.5±	0.122*; 0.463†; 0.312‡
E-cadherin	25.0±15.1	28.6±15.0	26.7±16.2	25.3±14.9	26.2±14.8	42.4±16.7	0.490*; 0.755†; 0.003‡
ZO-1	4.9±4.1	6.1±4.0	4.6±4.8	5.9±5.2	5.2±4.0	11.0±5.7	0.459*; 0.425†; <0.001‡
Mast cell	13.2±7.2	13.6±5.7	14.2±7.0	12.1±6.5	13.3±6.8	1.3±1.2	0.868*; 0.259†; <0.001‡
TUNEL	2.6±2.5	2.9±2.6	2.9±2.8	2.5±2.3	2.6±2.5	0.1±0.3	0.714*; 0.568†; 0.002‡

UUT: upper urinary tract; Upper UUT: upper 1/3 ureter or kidney; Lower UUT: middle or lower 1/3 ureter; G (+): patients with glomerulations; G (−): patients without glomerulations.

*: p values between G (+) and G (−) groups.

† : p value between lower and upper UUT groups.

‡ : p values between UUT urolithiasis patients and controls.

## Discussion

This study demonstrated that development of glomerulations after cystoscopic hydrodistension was highly prevalent in patients with UUT urolithiasis. Patients with middle or lower 1/3 ureteral stones had a smaller MBC and a higher glomerulation rate than those with upper 1/3 ureteral or renal stones. The immunohistochemistry study revealed that urothelial dysfunction characterized by increased apoptosis, inflammation and decrease of E-cadherin and ZO-1 expressions was found in the bladder mucosa of UUT urolithiasis patients.

This study also found that 53.8% of UUT urolithiaisis patients had LUTS and a smaller MBC. Although the immunohistochemistry study revealed no significant difference in the urothelial dysfunction and bladder inflammation between patients with and without LUTS, these findings suggest communication such as cross-talk between the UUT and bladder might exist. However, there was no significant difference in the immunohistochemistry findings between urolithiasis patients with and without glomerulations. This probably reflects the phenomenon that not all microscopic inflammation produces macroscopic change in the bladder.

Cross-organ sensitization between the lower gut and pelvic urinary or gynecologic organs is a common observation in animal models and human studies. [8] The urinary bladder may be more vulnerable to cross-modulation than other pelvic organs in cases of pelvic/lower abdominal cross-organ sensitization. The main mechanisms of cross-organ sensitization include peripheral and central pathways. [8], [9] Peripherally mediated cross-organ sensitization occurs upon the convergence of sensory inputs at the level of the dorsal root ganglion. Centrally mediated cross-organ sensitization is caused by the convergence of sensory inputs through the second order spinal neurons, which occurs in the spinal cord or brain. As a result of cross-organ sensitization, neurogenic inflammation develops in an adjacent organ.

Sensory input of the kidney from the visceral afferent fibers (accompanying the sympathetic fibers) retrogradely travels to T10- L1 levels of spinal cord through prevertebral and parapvertebral ganglia, and that of ureter to T11- L2 levels of spinal cord. [10] In urinary bladder, visceral pain fibers follow sympathetic fibers retrogradely to T11- L2 levels of spinal cord, and the parasympathetic fibers were originated from S2–S4 levels of spinal cord. The levels of afferent neurons of kidney, ureter, and bladder within spinal cord are overlapped. We hypothesized that stone in the UUT produced local inflammation, which resulted in neurogenic inflammation of the bladder via cross-talk sensitization. Consequently, the neurogenic inflammation produced changes of urothelial dysfunction and suburothelial inflammation within the bladder, which is one of the possible causes of glomerulations during cystoscopic hydrodistension. All of the eligible patients were free of urinary tract infection and pyuria, and that glomerulations were not directly caused by inflammation from urine within the urinary tract but by inflammation originating from the bladder wall itself was suggested. UUT urolithiasis patients with lower stone positions had a higher glomerulation rate than those with upper positions. The closer the UUT lesion to the bladder, the higher rate of cross-talk between them is likely to be the explanation.

We have previously found that urinary nerve growth factor levels increased in patients with urolithiasis as well as in overactive bladder and IC/BPS, suggesting some inflammatory process might be involved in the urinary tract with urolithiasis. [11] Neurogenic inflammation is triggered by the release of neurotransmitters that cause pain and inflammation from sensory nerve terminals. [12] Mast cells synthesize and release a number of vasoactive and chemotactic factors, and appear to play a role in neurogenic inflammation. [13], [14] Our results showed that suburothelial inflammation with mast cell infiltration was more significant in UUT urolithiasis patients than in control subjects. The results also correspond to the hypothesis of cross-organ sensitization of the UUT and bladder through the mechanism of neurogenic inflammation.

E-cadherin plays a critical role in cell-to-cell adhesion, and ZO-1 is recognized as a tight junction protein for maintaining the highly resistant urothelial barrier. [15] Evidence of the association of reduced E-cadherin expression and increased pain scores in patients with IC/BPS [3] and the establishment of a molecular connection between E-cadherin and TRPV4 [16] suggest that E-cadherin is associated with bladder sensation and barrier function. In addition, defective adhesive function (decreased E-cadherin expression) could lead to reduced bladder capacity. [6] In comparison with the data of IC/BPS patients in our previous study [6], UUT urolithiasis patients had similarly decreased adhesive and junction protein expression and a similarly smaller MBC, indicating similar down-stream urothelial dysfunction and inflammation between these two urinary tract diseases.

Our previous studies have shown that urothelial dysfunction, increased suburothelial inflammation and apoptosis were present in patients with ketamine-related cystitis [6], IC/BPS [3], overactive bladder [5], and recurrent urinary tract infection [17]. In the present study, UUT urolithiasis patients also had similar immunohistochemistry results. The underlying pathogenesis of these lower urinary tract disorders seem not the same, but neurogenic inflammation is likely to be one of the common pathophysiologies of IC/BPS [2], overactive bladder [5] and UUT urolithiasis, as shown in this study. Through the investigations of IC/BPS, urothelial dysfunction and suburothelial inflammation could interact and form a vicious cycle provoking and maintaining inflammatory reactions in the bladder. [2], [18] The co-existence of urothelial dysfunction, suburothelial inflammation, and apoptosis is not specific to IC/BPS, but is commonly found in the other lower urinary tract disorders which cause chronic inflammation of the bladder.

Development of glomerulations after cystoscopic hydrodistension is considered a prerequisite for the diagnosis of IC/BPS based on the National Institute of Diabetes and Digestive and Kidney Disease (NIDDK) criteria. [19], [20] However, bladder glomerulations is not only present in IC/BPS, but also in some pelvic organ diseases including urethral sphincter deficiency [21], endometriosis [22] or chronic pelvic pain [23]. Bladder glomerulations are also reported in asymptomatic women undergoing tubal ligation. [24] The specificity of glomerulations after cystoscopic hydrodistention and its pathophysiology are still unclear. According to NIDDK criteria, bladder stone and ureteral stone should be excluded before making of a diagnosis of IC/BPS. [19], [20] European Society for the Study of Interstitial Cystitis also mentioned that the diagnosis of IC/BPS was made on the basis of exclusion of confusable diseases, including bladder and lower ureteral stone. [25] The necessity of exclusion of bladder and ureteral stones in the diagnosis of IC/BPS is conceptualized but has not been proved. In addition, the role of renal stones in the exclusion of IC/BPS has not been established. This study revealed the percentage of glomerulations was high (56.5%) in the patients with renal stones. The high glomerulation rate in UUT urolithiasis patients strengthens the necessity of excluding urolithiasis at any site of UUT in the diagnosis of IC/BPS.

There are several limitations in the present study. First, among the patients undergoing bladder biopsies, the number of patients without glomerulations was relatively small. Second, the connection between UUT and lower urinary tract was hypothesized through immunohistochemistry study for neurogenic inflammation and lacked direct evidence. Third, the controls were all female patients, and there were few differences between sex in the clinical and immunohistochemistry results. In the future, more thorough design of animal or human study should be established to elucidate the complete mechanism and the corresponding changes from the molecular level to clinical presentation.

## Conclusions

The phenomenon of glomerulations after cystoscopic hydrodistention is not specific for IC/BPS. The incidence of glomerulations in UUT urolithiasis patients is high, especially in those with stones located in the middle or lower 1/3 ureter. Patients with upper 1/3 ureter or renal stones also had glomerulations after hydrodistenion, which strengthens the necessity of excluding urolithiasis at any site of UUT in the diagnosis of IC/BPS. Urothelial dysfunction, increased suburothelial inflammation, and apoptosis are observed in the bladder tissue of UUT urolithiasis patients, indicating the existence of cross-talk of inflammation between the UUT and lower urinary tract. Patients with UUT urolithiaisis concomitant with LUTS had a smaller MBC, which may explain the presence of irritative bladder symptoms.
